# Domain tethering impacts dimerization and DNA-mediated allostery in the human transcription factor FoxP1

**DOI:** 10.1063/5.0138782

**Published:** 2023-05-15

**Authors:** Perla Cruz, Nicolás Paredes, Isabel Asela, Narendar Kolimi, José Alejandro Molina, César A. Ramírez-Sarmiento, Rajen Goutam, Gangton Huang, Exequiel Medina, Hugo Sanabria

**Affiliations:** 1 Departamento de Biología, Facultad de Ciencias, Universidad de Chile, Las Palmeras 3425, Casilla 653, Santiago 7800003, Chile; 2 Department of Physics and Astronomy, Clemson University, Clemson, South Carolina 29634, USA; 3 Institute for Biological and Medical Engineering, Schools of Engineering, Medicine and Biological Sciences, Pontificia Universidad Católica de Chile, Av. Vicuña Mackenna 4860, Santiago 7820436, Chile; 4 ANID – Millennium Science Initiative Program – Millennium Institute for Integrative Biology (iBio), Santiago 7820436, Chile

## Abstract

Transcription factors are multidomain proteins with specific DNA binding and regulatory domains. In the human FoxP subfamily (FoxP1, FoxP2, FoxP3, and FoxP4) of transcription factors, a 90 residue-long disordered region links a Leucine Zipper (ZIP)—known to form coiled-coil dimers—and a Forkhead (FKH) domain—known to form domain swapping dimers. We used replica exchange discrete molecular dynamics simulations, single-molecule fluorescence experiments, and other biophysical tools to understand how domain tethering in FoxP1 impacts dimerization at ZIP and FKH domains and how DNA binding allosterically regulates their dimerization. We found that domain tethering promotes FoxP1 dimerization but inhibits a FKH domain-swapped structure. Furthermore, our findings indicate that the linker mediates the mutual organization and dynamics of ZIP and FKH domains, forming closed and open states with and without interdomain contacts, thus highlighting the role of the linkers in multidomain proteins. Finally, we found that DNA allosterically promotes structural changes that decrease the dimerization propensity of FoxP1. We postulate that, upon DNA binding, the interdomain linker plays a crucial role in the gene regulatory function of FoxP1.

## INTRODUCTION

Transcription factors (TFs) are proteins that modulate the gene expression by gating access to DNA in response to changes in the organization of the genome.^1,2^ To accomplish their function, TFs use their DNA-binding domain to target sequences with high affinity and specificity.^3–6^

In addition to their DNA-binding domains, most eukaryotic TFs use their multidomain architecture and large disordered regions to assemble into functional protein–protein and protein–DNA complexes.^7^ In many cases, the final transcriptional complex bound to DNA depends on TF dimerization,^8^ highlighting the intricate interaction between flexible and disordered regions, their multidomain architecture, and the function of eukaryotic TFs.

**FIG. 1.**
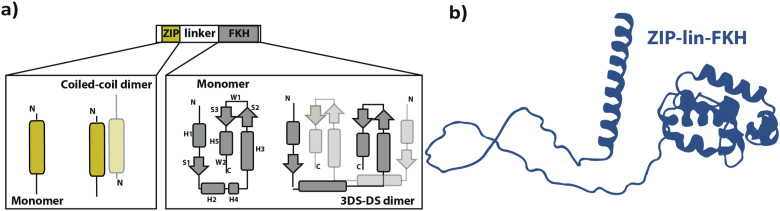
Domain topology and structure of human FoxP1. (a) A mostly disordered linker (lin) tethers ZIP (Leucine-Zipper) and FKH (Forkhead) domains in human FoxP proteins. Zooming into ZIP and FKH domains, we present the secondary structure topology (α-helix as round boxes, β-sheets as arrows, and coils as lines) and canonical dimerization mechanisms (antiparallel coiled-coil and three-dimensional domain swapping). (b) Tridimensional organization of the region ZIP-linker-FKH (ZIP-lin-FKH) in human FoxP1, as predicted by Alpha Fold.^24^

One of the most widespread eukaryotic TF domains is the leucine zipper (ZIP).^9–11^ ZIP domains belong to the basic leucine zipper (bZIP) superfamily, involved in different metabolic aspects, such as cellular proliferation, stress response, and homeostasis.^11–13^ ZIP domains are typically 30–80 amino acids long and can form coiled-coil helical dimers^9–11^ [Fig. 1(a)]. In addition, bZIP proteins can bind DNA as monomers or dimers,^14–17^ suggesting that ZIP domains regulate the formation of TF-DNA complexes.

The human Forkhead (FKH) Box P subfamily of TFs (FoxP 1, FoxP2, FoxP3, and FoxP4) is unique among the Fox family as they share a ZIP domain connected by an 80–100 residue long disordered region to the Forkhead (FKH) DNA binding domain.^18–20^ In addition, the isolated FKH domain of most FoxP proteins dimerizes *in vitro* via three-dimensional domain swapping (3D-DS) [Fig. 1(a)] and can adopt both monomeric- and dimeric complexes bound to DNA.^21–23^ The unusual architecture of FoxP proteins with two dimerization domains (ZIP and FKH domains) challenges the need for ZIP domains as dimerization domains but reinforces their role as regulatory domains.

The FoxP domain architecture offers novel insights into the function of ZIP domains and disordered linkers as allosteric modulators of interdomain communication. In the FoxP subfamily, an ∼90-residue-long flexible linker connects the ZIP domain to the Forkhead (FKH) domain. Additionally, both domains are known to regulate homo- and heterodimerization in cells,^25^ while isolated ZIP and FKH domains adopt dimeric structures.^21,22,26^ In human FoxP2 and FoxP3, ZIP domains modulate dimerization and DNA binding. In FoxP2, the presence of the ZIP domain increases the dimerization and DNA-binding kinetic rates,^27^ whereas in FoxP3, ZIP mutations impair dimerization and change gene expression.^28^ Moreover, heterodimerization between FoxP members in cells has only been observed in the ZIP domain.^25^

**FIG. 2.**
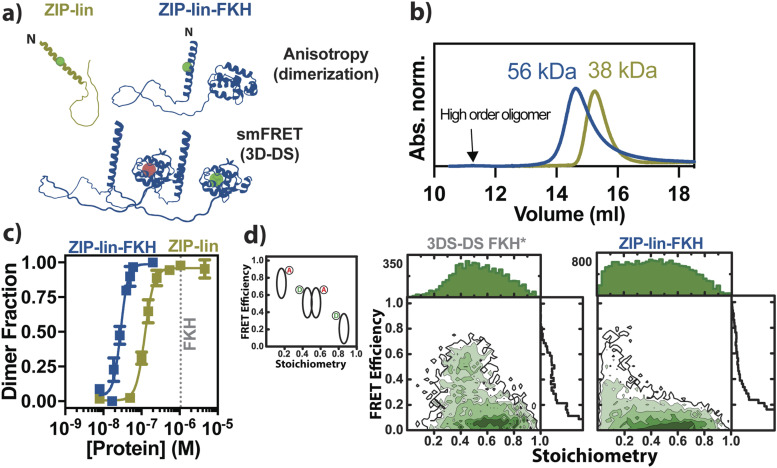
Dimerization analysis of ZIP-lin and ZIP-lin-FKH. (a) Labeling strategy to determine the dimerization of the proteins and the three-dimensional domain swapping (3D-DS) of the ZIP-lin-FKH. We monitored the changes in anisotropy of a BODIPY-labeled single-cysteine mutant of both ZIP and ZIP-FKH proteins (in green) in the same position (C356) in the titration experiments, whereas the 3D-DS ability of the ZIP-lin-FKH was evaluated by single-molecule FRET by following the FRET efficiency between the donor (ATTO488) and the acceptor (ATTO647N) in C492 in each monomer. (b) Experimental molecular weight estimation of ZIP (molecular weight: 17 kDa) and LZ-FKH (25 kDa) at 10 *µ*M of protein concentration. The value was extracted from a calibration curve using known proteins as reference (see the section titled “Material and Methods”). ZIP (olive) and LZ-FKH (blue) domains elute as a dimer. The arrow indicates the presence of a small population of high-order oligomers. (c) Dimer fraction comparison between ZIP and ZIP-FKH in each titration experiment. In both cases, 5 nM of each labeled protein was titrated with their respective unlabeled version. Each dimerization curve was measured in duplicate, showing the mean and its respective standard deviation in the text. The dissociation constant (*K*d) value of the isolated FKH is shown in gray, as previously reported.^32^ (d) (left) Schematics for the identification of the double-labeled dimer by single-molecule FRET efficiency vs stoichiometry. Both donor (D, green) and acceptor (A, red) labeled the 3D-DS FKH* (middle) and ZIP-lin-FKH(C493) (right) were combined to adopt the double-labeled dimer. The stoichiometry parameter is used to identify the double-labeled dimer. An unlabeled protein concentration of 10 nM ZIP-lin-FKH was used to maintain the dimeric state. The FRET efficiency obtained was compared with the previous 3D-DS dimer of the isolated FKH* domain previously reported.^33^ Correction parameters and specific protocol for dimerization can be found in the section titled “Material and Methods” and in the supplementary material.

However, it is unclear how dimerization and DNA binding of human FoxP proteins mediate the interdomain communication between ZIP and FKH domains. Here, we deciphered the consequences of tethering ZIP and FKH domains in FoxP1 by probing computationally [via replica exchange discrete molecular dynamics (DMD) simulations] and experimentally (ensemble and single-molecule fluorescence spectroscopy) the biophysical properties of two constructs encompassing the ZIP-lin region (residues 342–462) and ZIP-lin-FKH (residues 342–548) domains [Fig. 2(a)].

We determined that the dimerization of the isolated ZIP domain is more favorable than the isolated FKH domain. Interestingly, ZIP’s coiled-coil interactions stabilize ZIP-lin-FKH dimers and dramatically promote FoxP1 dimerization. We also predicted that, even under monomeric conditions, the ZIP domain has a high propensity for α-helical content, challenging the classical view of the disorder-to-order transition observed in ZIP coiled-coil dimerization.^15,29–31^

Finally, we analyzed the impact of the DNA when bound to the ZIP-lin-FKH construct by characterizing changes in the local behavior of the tethered domains. We found that the presence of the ZIP domain does not impact the affinity between FoxP1 and its cognate DNA, suggesting that the ZIP domain mainly serves as a dimerization-promoting domain. However, using single-molecule fluorescence anisotropy, our results indicate that binding to DNA causes local changes in both ZIP and FKH domains, supporting the idea of an allosteric interdomain communication in response to DNA binding. Moreover, we observed that FoxP1 is in dynamic exchange between two states, which we postulate corresponds to open and closed states reported by rxDMD simulations under monomeric conditions. Binding to DNA seems to stabilize the closed structure of FoxP1, impacting both ZIP and FKH domains, and corroborates the idea that the ligand exerts an allosteric regulation. Overall, our finding suggests that FoxP1 in cells will be in its dimeric form, and tethering the domains inhibits 3D-DS FKH dimerization. A step thought to be crucial as gene repressors.

## RESULTS

### The dimerization propensity of FoxP1 depends on the presence of the ZIP domain

Previous studies in FoxP proteins showed that the ZIP domain is responsible for dimerization.^18,25,27^ However, we and others have reported that the FKH domain in FoxP TFs can dimerize via domain-swapping.^21–23,32^ To evaluate the ZIP domain’s thermodynamic impact on the dimerization properties of FoxP1, we generated single-cysteine substitutions in the ZIP domain containing the linker (hereafter named ZIP-lin)—ZIP(A356C)-lin—or the construct containing both ZIP and FKH [ZIP(356)-lin-FKH] and labeled them with BODIPY FL [Fig. 2(a)]. We monitored fluorescence anisotropy changes as a dimerization reporter when titrating with their respective unlabeled wild-type construct. We titrated 5 nM of each labeled protein for these experiments using different concentrations of their respective unlabeled protein (0–5000 and 0–200 nM for ZIP-lin and ZIP-lin-FKH, respectively) at 37 °C. We observed, in both proteins, a transition in the relative anisotropy as a function of the protein concentration, indicative of a monomer–dimer equilibrium. To corroborate this assumption, we used Size Exclusion Chromatography (SEC) for both constructs (ZIP-lin and ZIP-lin-FKH). We inquired about the prevalence of possible oligomeric species in the solution. At these higher concentrations, we found that the dimer is the most probable population [Fig. 2(b)].

**FIG. 3.**
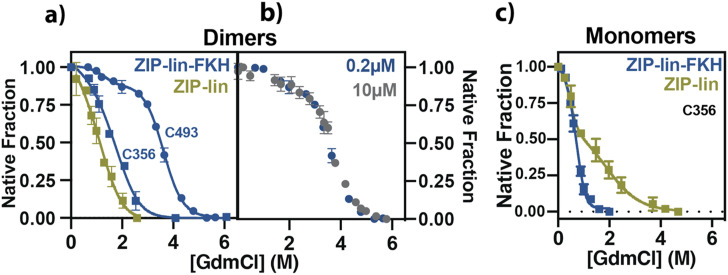
Stability and unfolding properties of ZIP-lin and ZIP-lin-FKH constructs. (a) Unfolding experiments using the isolated ZIP(C356)-lin (olive squares), ZIP(C356)-lin-FKH (blue squares), and ZIP-lin-FKH(C493) (blue circles). All the proteins were labeled with BODIPY-FL and used at 200 nM in each denaturant concentration. A two-state dimer ⇋ unfolded model was used to analyze the unfolding data for ZIP(C356) and ZIP(C356)-FKH, whereas a three-state dimer ⇋ intermediate ⇋ unfolded was used for the case of ZIP-FKH(C493). (b) Protein concentration effect on the unfolding transition of ZIP-FKH. The unfolding at 200 nM (a) was compared with the data using 10 *µ*M of unlabeled ZIP-FKH and monitored by tryptophan fluorescence. (c) Chemical unfolding assay for ZIP(C356)-lin (olive) and ZIP(C356)-lin-FKH (blue). The labeled proteins were maintained at 5 nM of protein concentration to favor the monomer. The unfolding of the monomer ZIP-lin was fitted to a three-state monomer ⇋ intermediate ⇋ unfolded mechanism (see the section titled “Material and Methods”). In contrast, the monomer ZIP-lin-FKH was fitted to a two-state monomer ⇋ unfolded mechanism. All unfolding experiments were carried out in duplicates. Means and standard deviations are plotted.

Next, we analyzed the dimerization isotherms using a two-state binding model (see the section titled “Data Analysis”), assuming that the anisotropy values reflect the dimer fraction [Fig. 3(b)]. Under this two-state model, the dissociation constants (*K*_*D*_) were 123 ± 10 and 29 ± 3 nM for the ZIP-lin and ZIP-lin-FKH constructs, respectively (Table S1). Similar studies with different bZIP transcription factors reported similar *K*_*D*_ values.^24,34^ In these cases, bZIP dimerization depends on the prior formation of monomer-DNA complexes.^14,16,30,31^ Interestingly, the observed behavior of both constructs suggests that the most likely state in the absence of DNA is the dimer, even with a low protein concentration.

Moreover, even when *K*_*D*_ of the ZIP-lin is significantly lower than for the isolated FKH domain [∼1 *µ*M, Fig. 2(b)],^32,33^ tethering both domains increases the equilibrium dimerization affinity by approximately sixfold.

To determine whether the *K*_*D*_ difference between ZIP and ZIP-FKH is due to the dimerization via 3D-DS at the FKH domains, we generated a single-cysteine mutant (C493) in the FKH domain. We labeled the ZIP-FKH(C493) construct with either ATTO488 or ATTO647N and mixed them to form a dimer. We selected this position to compare with previous single-molecule FRET experiments with the isolated FKH domain in conditions of the 3D-DS dimer.^33^

We analyzed the ZIP-FKH(C493) and FKH 3D-DS dimers using a two-dimensional FRET efficiency and stoichiometry [Fig. 2(d)] histogram. Although we observed dimers containing donor and acceptor dyes, these dimers display mostly low FRET efficiency [Fig. 2(d)]. When we compared these results with our previous observation of the FKH dimer^33^ [Fig. 2(d), left], our results indicated that the dimeric state of ZIP-lin-FKH does not form a 3D-DS dimer [Fig. 2(d)]. Hence, 3D-DS dimers depend on the tethering of the ZIP and FKH domains, and their dimerization mostly depends on ZIP-dependent coiled-coil interaction.

### Domain tethering stabilizes the ZIP-lin-FKH dimer

We determined that the dimerization propensity of FoxP1 mostly depends on ZIP-dependent coiled-coil interactions. However, domain tethering promoted dimerization. Therefore, we asked if the dimer stability depends on ZIP’s coiled-coil interactions.

To evaluate the conformational stability of both domains, we monitored the chemical denaturation of ZIP-lin and ZIP-lin-FKH dimers. Using a single-cysteine mutant in the ZIP-lin (C356) [Fig. 3(a)], we performed equilibrium unfolding studies with the ZIP-lin under dimeric conditions because *K*_*D*_ indicates that these constructs are dimeric at low concentrations. We then used 200 nM of BODIPY-labeled ZIP(C356)-lin incubated at different guanidinium chloride (GdmCl) concentrations and monitored the changes in fluorescence anisotropy as a reporter of domain unfolding.

We use a two-state thermodynamic unfolding model (N_2_ ⇋ 2U) to analyze the changes in fluorescence anisotropy relative to the dimer a 0M GdmCl [Fig. 3(a)]. We noticed a single transition at 0.5–1.5M of GdmCl. Using the mentioned unfolding model (see the section titled “Data Analysis”), we determined an unfolding free energy (*G*_*U*_) of 10 ± 0.5 kcal mol^−1^ for the ZIP-lin dimer, indicating that the domain-swapped dimer of the isolated FKH domain previously characterized (19 kcal mol^−1^)^32^ is ∼9 kcal mol^−1^ more stable.

To get insights into the combined stability of the tethered ZIP and FKH domains, we performed the same equilibrium unfolding studies for the ZIP-lin-FKH construct. With this construct, we independently monitored the stability of the ZIP and FKH domains by using the C356 mutant in the ZIP domain and a single-cysteine mutant in the FKH domain (C493). Both constructs were labeled with BODIPY FL. We measured the changes in the fluorescence anisotropy in the same denaturing conditions as for the ZIP-lin construct [Fig. 3(a)]. Again, we performed these experiments under dimeric conditions (200 nM) because of *K*_*D*_.

We observed distinct unfolding transitions for the two ZIP-lin-FKH constructs [blue curves in Fig. 3(a)]. Changes in the anisotropy at the ZIP domain [ZIP(C356)-lin-FKH] follow a similar two-state model as the ZIP(C356)-lin construct but with a slight shift toward higher concentrations of denaturant between 0.5 and 1.5M GdmCl. However, the unfolding behavior of the FKH domain [ZIP-FKH(C493)] shows two transitions. The first transition occurs in the same range of denaturant concentration as observed for the ZIP domain, and the second transition occurs between 2.5 and 4.5M of denaturant [Fig. 3(a)]. The folded fraction between 2 and 2.5M of denaturant is maintained at a native fraction of ∼0.85. We hypothesize that a near-native folded intermediate state is accumulated, while the ZIP domain unfolds. This behavior resembles the unfolding pathway of the isolated domain-swapped FKH domain,^35^ suggesting that the FKH domain unfolds under a three rather than a two-state model. Considering that both domains are tethered, our results indicate that the protein unfolds under a three-state unfolding reaction that involves the accumulation of an unfolded ZIP domain intermediate with almost no loss of the native structure at the FKH domain.

Even when we showed that the ZIP-lin-FKH dimer does not exhibit the canonical 3D-DS dimer at the FKH domain, we asked if the FKH domain could dimerize the ZIP-lin-FKH construct via a different pathway. In this scenario, the dimer’s dissociation could be observed by monitoring the unfolding of the FKH domain. Moreover, the unfolding transition of the FKH domain should depend on the protein concentration. In other words, the dissociation equilibrium will be unfavored, and the dimer will be more stable at the higher denaturing concentrations.^36^ Therefore, to determine the effect of the protein concentration in the FKH unfolding transition, we monitored the changes in the intrinsic fluorescence of the wild-type ZIP-lin-FKH at 10 *µ*M. Note that the FKH domain is the only region that contains tryptophan residues in this construct. We found that increasing 50 times the protein concentration from 200 nM to 10 *µ*M did not change the unfolding transition of the FKH domain [Fig. 3(b)], indicating that the dissociation of the protein is coupled to the unfolding of the ZIP domain. Thus, the unfolding pathway of the dimeric ZIP-lin-FKH must follow the three-state model, such as N_2_ ⇋ 2I^ZIP unfolded^ ⇋ 2U, where N_2_ is the dimer; I^ZIP unfolded^ is the unfolded intermediate where each construct has ZIP domain unfolded with the FKH partially folded (ZIP^unfolded^-lin-FKH^∼folded^); and U is when both domains are unfolded.

Considering this three-state unfolding mechanism of the ZIP-lin-FKH dimer, we analyzed the unfolding isotherms of the dimer with a two-state mechanism for the ZIP(C356)-lin-FKH dimer and a three-state model for the ZIP-lin-FKH(C493) dimer [Fig. 3(a); see the section titled “Data Analysis”]. We determined that the domain specific *G*_*U*_ for the ZIP is 12 ± 0.4 kcal mol^−1^, whereas *G*_*U*_ for the FKH are 12 ± 0.6 (N_2_ ⇋ 2I) and 6 ± 0.1 (I ⇋ U) kcal mol^−1^ (Table S2), corresponding to the two distinct transitions in the three-state model (Materials and Methods). Therefore, tethering these domains (i) increases the stability of the ZIP domain in ∼2 kcal mol^−1^, and (ii) the ZIP-lin-FKH dimer is stabilized by coiled-coil interactions of the ZIP domains.

Moreover, when we compared it with the previously determined unfolding mechanism of the 3D-DS dimer of the isolated FKH domain,^32,35^ we found that tethering the FKH with the ZIP domain increases around 4 kcal mol^−1^ the dissociation step of the FKH domain but has no impact on the stability of the partially unfolded intermediate ZIP^unfolded^-lin-FKH^∼folded^ [Fig. 3(c)].

Next, we asked whether the dimerization or the presence of the FKH domain is the main event stabilizing the ZIP domain. We monitored the local unfolding following changes in anisotropy in the monomeric form of the ZIP(356)-lin and the ZIP(C356)-lin-FKH construct. To ensure monomeric conditions, we performed these experiments using only 5 nM of the labeled construct [Fig. 3(d)]. Interestingly, the denaturation behavior of the monomeric ZIP-lin is different from the monomeric ZIP-lin-FKH. In this case, the ZIP-lin displayed a three-state unfolding mechanism, whereas the ZIP-lin-FKH changed from a three-state in the dimeric form to a two-state unfolding transition in its monomeric form.

Additionally, the total stability of the isolated ZIP-lin is 4 ± 1 kcal mol^−1^, whereas the stability of the ZIP tethered to the FKH is 6 ± 1 kcal mol^−1^ (Table S3). Compared with the dimeric analysis, the ZIP’s stability does not change significantly by the dimerization, but the FKH impacts its stability. These results suggest that tethering both domains (ZIP-lin-FKH) stabilizes the ZIP domain instead of dimerization-induced stability.

### Domain tethering in the monomer increases the stability of the ZIP domain

In the section “Domain tethering stabilizes the ZIP-lin-FKH dimer,” we determined that tethering the FKH to the ZIP-lin stabilizes the dimer by increasing the monomer’s stability. One question arises about the structural organization of ZIP-lin-FKH in its monomeric form and if contacts between the ZIP domain and the FKH domain can explain the increased stability.

To obtain a detailed structural interpretation of this stabilization, we performed replica exchange Discrete Molecular Dynamics (rxDMD) simulations^37^ using the ZIP, the ZIP-lin, and the ZIP-lin-FKH constructs and explored their energy landscape. These simulations have been extensively used to describe interdomain communication^38–40^ and disorder behavior.^39,41–43^

**FIG. 4.**
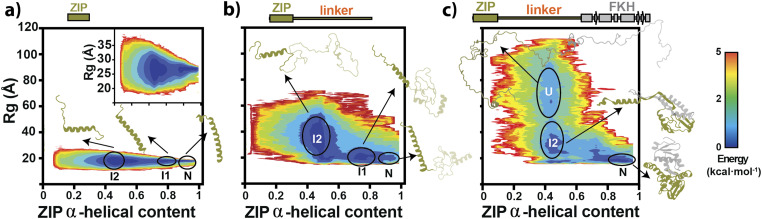
rxDMD simulations for monomeric FoxP1 ZIP, ZIP-lin, and ZIP-lin-FKH. Energy landscape [Potential Mean Force (PMF)] against *R*_*g*_ and α-helical content of the different ZIP constructs: ZIP domain alone (a), ZIP-lin (b), and ZIP-lin-FKH (c), showing the energy minima for the predicted native (N), intermediates (I, 1, and 2), and the unfolded (U) states. A representative structure is shown for each energy minimum (basin) (see the section titled “Material and Methods”). For easy understanding, the ZIP-linker region is colored in olive, whereas the FKH domain is colored in gray. The inset in (a) corresponds to the zoom of the ZIP’s PMF plot to better show the energy minima using the same x axis scale.

We plotted the Potential Mean Force (PMF) along two reaction coordinates: the *R*_*g*_ and the α-helical content of the ZIP domain in all simulated constructs (Fig. 4). In all cases, we performed the analysis at 300 K.

The ZIP domain, with and without the linker, showed two energy minima with a small energy barrier between them at high α-helical content and similar *R*_*g*_ [Figs. 4(a) and 4(b)], which can be described as the native and an intermediate state (I1). Additionally, we found a third energy minimum in the ZIP construct alone that does not increase *R*_*g*_, but it is expanded when connected to the linker. We defined this minimum as a second intermediate (I2). By inspecting the three basins, we randomly extracted a representative configuration for each defined basin [Figs. 4(a) and 4(b)], specifying the possible configurations by restricting the search between ±5 Å of the mean *R*_*g*_ and ±0.02 of mean α-helical content. We found a loss of an α-helical content in the N-terminal region for the ZIP and ZIP-lin in their corresponding I1 minimum. In contrast, in I2, mostly the ZIP and ZIP-lin constructs are unfolded at the C-terminal region [Figs. 4(a) and 4(b)]. Therefore, the extension of the linker explains the observed differences between *R*_*g*_ in the I2 basin between the ZIP and ZIP-lin. These results suggest that the linker does not impact the structure and behavior of the ZIP domain.

Next, we inspected the structure in the ZIP domain using the ZIP-lin-FKH construct [Fig. 4(c)]. We noticed that most of the energy minima at high α-helical content falls in the same region of the native (N) ZIP and ZIP-lin [Fig. 4(c)], suggesting that this state is favored in the ZIP domain when tethered to the FKH domain. In addition, we identify a second energy minimum with higher *R*_*g*_ and loss of α-helical content that falls in the same region of the I2 basin observed for ZIP and ZIP-lin.

Using the same clustering selection, we observed that the main difference between basins N and I2 found in ZIP-lin-FKH could be explained, besides the local unfolding of the ZIP domain, by the loss of interdomain association [Fig. 4(c)]. For example, in the predicted native state, the ZIP and FKH domains have short interdomain distances. However, interdomain contacts are significantly lost during the unfolding transition. Moreover, the increase of the energy barrier between the N and I2 states suggests that tethering the FKH domain to the ZIP domain stabilizes the ZIP domain, as observed in our experimental unfolding experiments. Finally, the third basin found (U) in the ZIP-lin-FKH construct [Fig. 4(c)] best describes an unfolded ensemble since most of the secondary structure is lost.

Overall, rxDMD simulations predict that tethering the FKH domain to the ZIP domain allows for an interdomain communication that can be regulated by exchanging between a closed (N) state—where the FKH and ZIP domains share interdomain contacts—and an open (I) state—where interdomain contacts are lost.

### DNA-mediated structural allostery between the ZIP and FKH domains modulates dimerization

We determined that, although the presence of the ZIP domain is required for FoxP1 dimerization, the presence of the FKH domain increases its conformational stability. Moreover, rxDMD evidences a possible interdomain communication. We hypothesized that ligand interactions could allosterically regulate interdomain communication. Thus, we inquired about the DNA binding properties of the ZIP-lin-FKH dimer and how this binding could impact each domain.

**FIG. 5.**
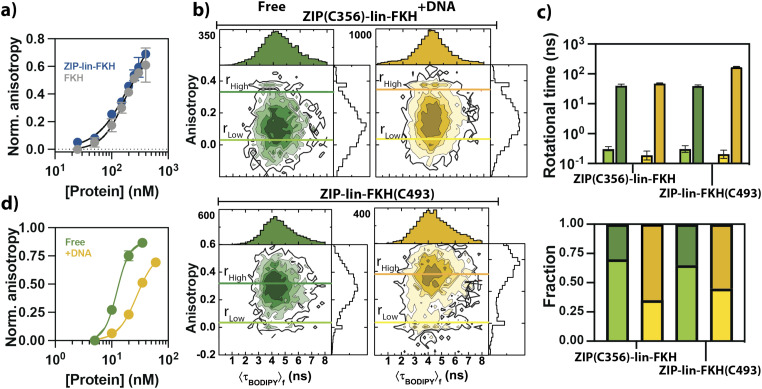
Allostery effects in the ZIP and FKH domains upon binding to DNA. (a) DNA binding assays with the isolated FKH (gray circles) or the ZIP-FKH (blue circles). A labeled DNA ligand (see the section titled “Material and Methods”) was incubated with different concentrations of the specified wild-type protein (ZIP-lin-FKH and FKH). The DNA’s fluorescence anisotropy was determined at 525 nm upon excitation at 480 nm. Fluorescence anisotropy values are normalized to the value without protein. (b) Two-dimensional histogram of *r*_*G*_ vs ${\left\langle \tau_{\mathrm{BODIPY}}\right\rangle }_{f}$ for the ZIP(C356)-lin-FKH and ZIP-lin-FKH(C493) BODIPY labeled constructs under free or DNA-bound conditions. The dark and light lines represent the sample’s high and low anisotropy extracted by fitting the data using a time-resolved analysis with two rotational correlation components, giving high (*r*_*High*_) and low (*r*_*Low*_) anisotropies. (c) Rotational times and its respective fraction for each anisotropy component and respective sample (*r*_*High*_ and *r*_*Low*_). Dark and light bars represent the high and low anisotropy components under free (green) and DNA-bound (yellow) conditions, as determined from the Perrin equation (see the section titled “Material and Methods”). (d) Dimerization analysis with DNA. 5 nM of the labeled ZIP-lin-FKH(C492) monomer was titrated with different concentrations of wild-type ZIP-lin-FKH without (free) or with 10 nM of unlabeled DNA. Both assays were normalized to the fluorescence anisotropy of the labeled protein without the unlabeled protein. The titration curves were carried out in duplicate. Means and standard deviations are plotted.

Using a labeled version of the cognate DNA sequence recognized by the FKH of FoxP1, we performed DNA–protein binding experiments with increased concentrations of the ZIP-lin-FKH and the FKH constructs. We monitored changes in the anisotropy of the labeled DNA as a function of the added protein [Fig. 5(a)]. Surprisingly and different from the observed effect in other FoxP proteins,^20,27^ the DNA-FKH affinity of FoxP1 is primarily independent of the presence of ZIP, with *K*_*D*_ values of 170 ± 20 and 176 ± 40 nM for the ZIP-lin-FKH construct and the isolated FKH domain, respectively.

Therefore, tethering the ZIP domain to the FKH domain has no impact on the FKH domain’s ability to bind DNA. Next, we wanted to probe the impact of DNA binding in both domains and asses possible allostery when forming protein–DNA complexes. We used the BODIPY-labeled ZIP(C356)-lin-FKH and ZIP-lin-FKH(C493) constructs. These constructs have the dye at the different domains, and thus, fluorescence anisotropy will report on the domain behavior with (+) and without (free) DNA. We performed these experiments at the single-molecule level (Tables S3 and S4). We first determined possible changes in the fluorescent properties of the dye by examining the fluorescence lifetime and intensity of the labeled proteins (Fig. S2). For both proteins, the changes of these fluorescent properties are less than ±10% in the presence of the DNA, ruling out significant photophysical effects, such as PIFE (protein induced fluorescence enhancement)^44^ or significant static and dynamic quenching.

In single-molecule Fluorescence Anisotropy (smFA) experiments, the labeled constructs are at picomolar concentration and excited under polarized light, and two channels detect the polarized emission.^41^ However, we studied the labeled constructs under dimeric conditions by adding ∼200 nM of unlabeled wild-type construct [Fig. 5(b)]. We correlated the fluorescence anisotropy (*r*_*G*_) and the average fluorescence lifetimes ${\left\langle \tau_{\mathrm{BODIPY}}\right\rangle }_{f}$ of individual molecules in two-dimensional histograms [Fig. 5(b)]. Note that the fluorescence anisotropy reports on the rotational properties of the molecule and the complex. Therefore, differences in the fluorescence anisotropy report on conformational changes.^41,45,46^ Moreover, because we measured the smFA experiments under confocal illumination, we corroborated the ZIP-lin-FKH:DNA complex formation by determining the characteristic diffusion time of the labeled constructs without and with DNA (Fig. S1).

In the two-dimensional histograms [Fig. 5(b)], we observed ${\left\langle \tau_{\mathrm{BODIPY}}\right\rangle }_{\;f}$ values centered between 4.2 and 4.5 ns and *r*_*G*_ values ranging from 0.1 to 0.4. Without DNA, both constructs show a single distribution in their *r*_*G*_. The construct that reflects the anisotropy at the ZIP domain peaks at ∼0.1, while the one reporting on the FKH domain peaks at ∼0.3. Interestingly, DNA binding has a differential impact depending on the observed domain. For example, the anisotropy distribution of the construct that reports on the ZIP domain slightly shifts to higher *r*_*G*_ with DNA compared to when it is free. However, the behavior of the fluorescence anisotropy that reports on the FKH domain has a pronounced effect. With DNA, two populations are evident with apparent *r*_*G*_ values of ∼0.05 and ∼0.4. These values inform us that the constructs are in dynamic exchange between low and high anisotropy values and that the ZIP and the FKH domains have different behaviors when bound to DNA.

Next, we evaluated the sub-ensemble time-resolved anisotropy decays using one or two rotational correlation times (*ρ*) (Data analysis) and two fluorescence lifetimes. This procedure allows us to determine the dye’s rotational properties and estimate different anisotropy components with their respective population fraction. We fitted the sub-ensemble time-resolved anisotropy decays under all conditions using two rotational times (*ρ*) instead of one (Table S5). We obtained a fraction with a low average anisotropy (*r*_*G*_) between ∼0.01 and 0.02 and a high *r*_*G*_ between ∼0.3 and 0.35 [Figs. 5(c) and S4] for both constructs. Analysis of the *ρ* revealed a low *ρ* between 0.1 and 0.3 ns and a high *ρ* between 40 and 170 ns depending on whether it is free or bound to DNA [Fig. 5(c) and Table S3]. This behavior is consistent with the 2D histograms, where we note that the peak of the distribution falls within the limits of the high and low anisotropy values. Hence, it suggests that the low *ρ* corresponds to when the dye is free to rotate even when attached to the protein’s backbone, hence reports on the local anisotropy. The slow component can be interpreted as the global protein’s rotation or global anisotropy component.^47,48^

We observed that DNA binding does not change the fluorescence lifetime or the intensity of the dye (Fig. S2); consequently, the high *ρ* suggests that there is a significant change in the hydrodynamic radius, and because $\rho \gg {{\langle \tau }_{\mathrm{BODIPY}\;}\rangle }_{\;f}$, we can safely ignore photophysical effects, such as quenching. In this scenario, our results suggest that both domains exhibit dynamic exchange between at least two different conformations with different rotational correlation times, consistent with the qualitative assessment of the two-dimensional histograms.

Moreover, we determined the fraction of molecules characterized by the low or high *ρ*. Without DNA, both constructs have a high population fraction (∼70%) that exerts a low *ρ* [Fig. 5(c)]. Hence, both domains exhibit similar behavior. However, when bound to DNA, both constructs exhibit two significant differences in the fluorescence anisotropy. First, high *ρ* of the FKH increases significantly compared to any of the other conditions [Fig. 5(c), top]. Second, the relative contribution of the high rotational reaches ∼60–70% for both constructs. Therefore, we can confidently conclude that there is an allosteric effect between the ZIP and FKH domains when FoxP1 binds to DNA. This interaction differently impacts the mobility of the FKH domain [Fig. 5(c), bottom].

Finally, we calculated if the observed DNA-induced interdomain allostery negatively impacts the dimerization of FoxP1. We repeated the dimerization assay shown in Fig. 2 but using the monomer labeled ZIP(C356)-lin-KFH monomer incubated with DNA as the starting point. By titrating this complex with different unlabeled wild-type ZIP-lin-FKH, we followed the changes in anisotropy. In this case, the unlabeled protein was previously denatured to ensure the monomeric form upon the addition. Interestingly, preincubating the labeled monomer with the DNA decreases the dimerization affinity by twofold, obtaining an apparent *K*_*D*_ of 55 ± 6 nM. Altogether, our results indicate that DNA promotes interdomain allostery that differentially impacts the tethered ZIP and FKH domains and decreases the dimerization propensity of FoxP1.

## DISCUSSION

We studied how tethering the ZIP and FKH domains in FoxP1 modulates the thermodynamic stability, dimerization, and DNA binding for each domain. These processes are crucial aspects of the function of transcription factors. We chose the human FoxP subfamily, particularly FoxP1, as a model system because these proteins shared two dimerization domains (ZIP and FKH) tethered by a disordered linker. For example, given that these two domains have a redundant dimerization function, whether the ZIP and FKH domains behave as a single cooperative unit was unknown.

Our results showed that these domains, when tethered, do not behave as a cooperative dimerization unit because the dimerization relies on the ZIP domain’s stability (Fig. 3). We also observed that tethering these domains inhibits the 3D-DS dimerization of the FKH domain (Fig. 2).

Moreover, the FKH domain, when tethered to the ZIP domain, stabilizes the ZIP domain and, more importantly, increases the stability of the ZIP-lin-FKH dimer. This observation is unique because other protein models suggest that each domain’s stability and folding mechanism are unaffected when tethering dimerization domains,^49,50^ thus the importance of interdomain communication in understanding protein’s functions.^38,51,52^ Specific to human FoxP proteins, others have highlighted the importance of the ZIP domain in FoxP dimerization.^18–20,26,27,53^ However, our results provide an additional view shedding light on how domain tethering changes the stability and dimerization of each domain and enables interdomain communication.

**FIG. 6.**
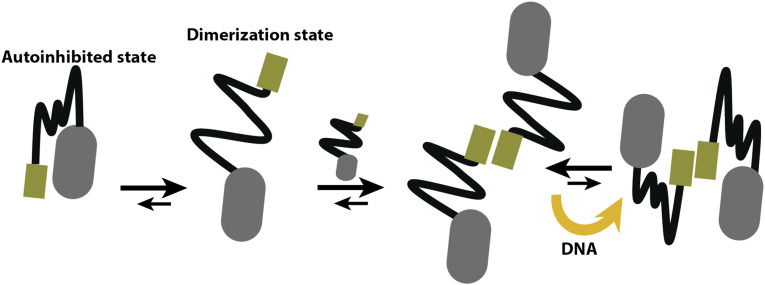
A proposed model of the impact of the tethering of ZIP-lin-FKH in protein properties. By our experimental and rxDMD, we predict that the equilibrium between two major conformations determines the ability of FoxP1 to dimerize. The closed conformation (high rotational correlation time in smFA) acts as an autoinhibited state for dimerization. The loss of interdomain contacts should be required to allow for coiled-coil dimerization. This open-closed equilibrium is maintained under dimeric conditions, and the presence of DNA stabilizes the dimer by increasing the closed conformation.

Molecular dynamic simulations predicted that the long and disordered linker could modulate the exchange between open and closed conformations in the monomeric form of the ZIP-lin-FKH construct. These closed- and compact-states are the first evidence of interdomain communication and provide clues on the possible regulatory role in FoxP1 dimerization. For example, the linker could act as an allosteric inhibitor of dimerization (Fig. 6). However, as the observed *K*_*D*_ for ZIP-lin-FKH suggests, the open conformation seems to be the predominant state in FoxP1, thus allowing for the accumulation of a stable dimeric protein.

Interestingly, the smFA experiments with the ZIP-lin-FKH construct support the view of dynamic equilibrium between open and closed dimers. In both domains, the slow rotational correlation time could report on the local stability of each domain because BODIPY has a smaller linker and longer lifetime compared with other dyes. Thus, BODIPY is more sensitive to structural changes than other dyes. Moreover, the fast rotational correlation time can be seen as a change to the secondary structure or stability loss. This interpretation has been used to understand the local stability of other proteins.^41,45,46^ Therefore, the slow rotational correlation time could represent the closed state predicted in rxDMD simulations, whereas the fast rotational time could represent the open, more flexible state.

Under this assumption, three reasons support the relationship between anisotropy and conformational changes. First, the fractional contribution of the fast rotational correlation time is predominant in both proteins without DNA, suggesting that the open state is highly populated, thus favoring the dimerization of FoxP1. Second, DNA binding has a differential effect on the ZIP and FKH domains. For example, DNA binding decreases the fractional contribution of the fast rotational correlation time in both domains but significantly increases the slow rotational correlation time on the FKH domain, not observed for the ZIP domain. In this case, we suspect that the FKH domain is prone to higher stability. Third, the presence of DNA decreases the dimerization ability of ZIP-lin-FKH, which is in line with the increased fraction of the closed state (Fig. 6).

Multidomain proteins point to the crucial role of disordered linker regions. These flexible linkers increase conformational entropy and decrease the energy cost for association and folding.^29,54,55^ Additionally, disordered linkers tether folded domains and promote interdomain interactions by increasing the effective concentration of the domains.^38,51^ A key modulating aspect of disordered linkers is their susceptibility to post-translational modifications, which can severely impact their flexibility and interdomain communication. The linker between ZIP and FKH domains in human FoxP3 contains conserved phosphorylation and acetylation sites with functional consequences.^26,56,57^ We suspect that the presence of this region is determinant not only in promoting dynamics but has the potential in promoting dimerization. Thus, post-translational modifications, such as phosphorylation and acetylation, could have a direct regulatory function.

Overall, we conclude that tethering the ZIP and FKH domains via a flexible linker enables interdomain communication, significantly impacting each domain’s dimerization ability and the allosteric response upon binding to DNA.

## MATERIAL AND METHODS

### Protein expression and purification

The Forkhead (FKH) and leucine-zipper (ZIP) domains, the region containing both domains (ZIP-FKH) of the human FoxP1, and their mutants (A356C, N493C) were cloned into a modified pET-28a vector containing a His6-tag, a TEV cleavage site, and an S-tag sequence toward the 5′ end of the gene. The plasmid containing FoxP1 mutants was created by PCR mutagenesis using the QuickChange Site-directed mutagenesis kit (Stratagene, La Jolla, CA, USA). The pET 28a vector was transformed into *E.coli* C41 cells (Invitrogen) to overexpress the FoxP1 protein. The protein expression was induced by 0.5 mM IPTG followed by overnight incubation at 37 °C for 6 h to attain the optical density at A_600_ in the range of 0.8–0.9. Furthermore, proteins were purified using Ni^2+^-NTA affinity chromatography, as described previously.

### Labeling of proteins

Different single-cysteine mutants were expressed and purified for labeling with BODIPY. Prior to labeling, 100 *µ*M of each protein [ZIP(C356)-lin, ZIP-lin-FKH(C493), and ZIP(C356)–FKH] was added to buffer A (20 mM Hepes, pH 7.8, 150 mM NaCl, 2M GdmCl) with 0.5 mM tris (2-carboxyethyl) phosphine hydrochloride (TCEP) and incubated over 30 min at room temperature. Next, we buffer exchanged by adding 500 *µ*l of the specified protein with 2 ml buffer A to the PD 10 column and further concentrated. The eluted protein was labeled with BODIPY-FL 488 by adding a five-fold excess of cysteine concentration, incubated for 2 h at room temperature, and finally 16 h at 4 °C. We carried out a buffer exchange the next day and measured the labeled protein concentration. Finally, the excess free fluorescent dye was removed by size exclusion chromatography.

### Size exclusion chromatography

Each labeled protein was separated from free dye using an HPLC system with a Superdex 75 increase column (Cytiva). First, the column was equilibrated with 50 ml of standard buffer (20 mM HEPES pH 7.8, 150 mM NaCl). Next, the labeled samples were eluted within one column volume using the same buffer. The collected fractions were stored at 4 °C for the experiments. For the case of unlabeled ZIP-lin and ZIP-lin-FKH proteins, an equilibrated aliquot of each construct was run using the same mentioned buffer.

### Dimerization, DNA-binding, and unfolding experiments

The experiments were carried out using the BODIPY—labeled ZIP(C356)-lin and ZIP(C356)-lin-FKH at 5 nM. The specified labeled protein was titrated using its wild-type version (ZIP-lin or ZIP-lin-FKH) from 0 to 5000 nM, and the samples were incubated at 37 °C before the anisotropy determination. Changes in anisotropy were analyzed to determine the dissociation constant (*K*_*D*_). For the case of DNA binding, a fixed concentration of 200 nM of purified labeled DNA was titrated with either wild-type FKH or ZIP-lin-FKH in a range of 0–500 nM. The binding process was followed by changes in anisotropy at 525 nm under an excitation of 480 nm. Finally, the dimerization experiment with DNA was carried out under the same conditions (5 nM of labeled protein) but before the titration, the monomer was previously incubated with 10 nM of DNA.

For the case of dimeric unfolding experiments, 200 nM of each labeled protein or the wild-type ZIP-lin-FKH at 10 *µ*M was incubated with different concentrations of either guanidine chloride (GmdCl). For the BODIPY-labeled samples, the unfolding transition was followed by anisotropy, as mentioned in the previous section, whereas for the wild-type ZIP-lin-FKH, the unfolding was followed by intensity changes at 323 nm under excitation at 295 nm. For the BODIPY-labeled samples [ZIP(C356)-lin and ZIP-lin-FKH(C493)], a 5 nM of labeled ZIP-lin or ZIP-lin-FKH was incubated with different GmdCl concentrations, and the unfolding transitions were analyzed as described for the labeled dimers.

### Replica-exchange discrete molecular dynamics (DMD) simulations

ZIP, ZIP-lin, and ZIP-lin-FKH domain structures were generated using Alphafold.^24^ In our experiments, we used all-atom replica exchange simulations in the DMD software package. For all structures, DMD equilibration was carried out with 500 000 ps each with ∼2 fs time step. DMD simulations were used to run inter-atomic forces in the all-atom model. In DMD simulations, we used the medusa force field with an implicit solvent model (CHARMM19-based energy function and Gaussian solvent-exclusion model) and discretized potentials as reported previously.^39–41^ Our experiments were carried out in a total of 18 replicas in which the molecule is allowed to exchange between a total of 18 different temperature baths, which are equally spaced from 280 to 370 K with a constant temperature using Andersen thermostat.^58^ DMD simulations were carried out for 500 ns for all constructs. Following it, the analyses were carried out using PyMoL and Python.

Representative structures from the 18 replicas were selected by identifying local minima (basins) in the PMF shown in Fig. 4 and by setting specific selection criteria on Rg $\pm 5\overset{{}^{\circ}}{A}$ and α-helical fraction ±0.02. Selected representative structures were chosen from at least 10 000 steps with 0.5 ns in separation between each representative snapshot. A single snapshot was used for visualization purposes.

### Ensemble and single-molecule anisotropy measurements

All ensemble BODIPY labeled single-cysteine mutants, and the labeled DNA was measured in a Jasco FP-8300 fluorimeter equipped with excitation and emission polarization filters. The anisotropy changes were determined by an excitation of 480 nm and the emission intensity between 500 and 600 nm, following the parallel and perpendicular polarized intensities at 519 nm for the excitation polarizer for the case of BODIPY-labeled protein and at 525 nm for the case of Alexa488-labeled DNA. The G factor was measured using free BODIPY dye at 200 nM concentration.

Single-molecule fluorescence anisotropy was carried out in a Zeiss LSM710 confocal system coupled with a diode laser at 485 nm (LDH-P 485 PicoQuant, Germany, power at objective 120 *µ*W). The freely diffused molecules excited and passed through a 40X detection volume, and the emitted fluorescence signal was collected with a 70-*µ*m pinhole using a 1.2 NA collar (0.17) corrected Zeiss objective. The signal was used through a bandpass HQ 520/35 filter, and the channel was further divided into parallel and perpendicular polarizer components. Two synchronized input channels and time-correlated single-photon counting (TCSPC) modules (MultiHarp 150P, PicoQuant, Germany) were used for data registration. Sub-ensemble time-resolved fluorescence data were collected as described previously.

All smFA experiments were carried out at 100–200 pM of labeled ZIP(C356)-lin-FKH or ZIP-lin-FKH(C493) diluted in the standard buffer in the absence or presence of DNA, combined with 200 nM of unlabeled protein, ensuring dimeric conditions as predicted by their *K*_*D*_. For smFA, we used a 500 *µ*l sample volume containing NUNC chambers (Lab-Tek, Thermo Scientific, Germany). The instrument response function (IRF) was determined by measuring water, whereas standard buffer for background subtraction and Rhodamine 110 for calibrations and G factor calculation were used.

### Single-molecule fluorescence correlation spectroscopy (smFCS)

Single-molecule fluorescence correlation spectroscopy was accomplished by selecting the single-molecule burst to differentiate the fluorescence photons from background photons. Then, the fluorescence species was autocorrelated based on the detection spectral windows to generate the respective correlation curves ($G\left(t_{c}\right)$) in the absence and presence of DNA. The diffusion time (*t*_*diff*_) was calculated using a global analysis as previously performed.^33^

## DATA ANALYSIS

### Equilibrium dimerization/DNA binding

All the dimerization and protein-DNA binding reactions were fitted into a two-state equilibrium, where the observed anisotropy (*r*_*obs*_) represents a fraction-weighted value of the true anisotropy of either the monomer (or free protein) or dimer (or protein-DNA complex). In that scenario, the dissociation constant (*K*_*D*_) is obtained from the following equation:

$$r_{\mathrm{obs}}=\frac{r_{max}\cdot \left[P\right]}{K_{d}+\left[P\right]}.$$ (1)

In this equation, *r*_*max*_ correspond to the amplitude of the initial and final anisotropy and [P] is the total protein. We transformed *r*_*obs*_ to dimeric fraction, considering the initial (monomeric state) and *r*_*max*_ (dimeric) values.

### Equilibrium unfolding

All the unfolding reactions were analyzed under a two-state (monomer or dimer) or three-state (monomer or dimer) mechanism depending on the protein. Considering these mechanisms, the unfolding free energy change (*G*_*U*_) will be, in all cases,

$$\Delta G_{U}=\Delta G_{\mathrm{H20}}^{0}+m\cdot \left[D\right],$$ (2)

where Δ*G*_*U*_ at certain denaturant (*D*) concentration depends on the intrinsic free energy change ($\Delta G_{\mathrm{H20}}^{0}$) in the absence of the denaturant and on the *m*-value, the empirical slope of the unfolding reaction. The observed signal (*S*_*obs*_) at the specified denaturant concentration will account for the intrinsic signal species (*S*_*N*_ and *S*_*U*_) and their respective fractions, as shown in Eq. (3) for a two-state system and in Eq. (4) for a three-state system, considering the intermediate signal (*S*_*I*_),

$$S_{\mathrm{obs}}=S_{N}\cdot f_{N}+S_{U}\cdot f_{U},$$ (3)

$$S_{\mathrm{obs}}=S_{N}\cdot f_{N}+S_{I}\cdot f_{I}+S_{U}\cdot f_{U}.$$ (4)

Moreover, the native fraction at the specified denaturant concentration can be represented for the dimer two-state dimer and a monomer.^59^ In the same context, the dimer and monomer three-state unfolding mechanism at the specified denaturant concentration can be analyzed as previously described.^36,60^

For all cases, the fitting process will give the unfolding equilibrium constant, by which the *G*_*U*_ value can be directly calculated.

Fitting procedures were performed using the software GraphPad Prism 8.0 (www.graphpad.com).

### Time-resolved fluorescence analysis

Time-resolved fluorescence decays [*F*(*t*)] were described using a multi-exponential model [Eq. (7)],

$$F_{norm}\left(t\right)=\sum_{i}^{n}x_{i\;}exp\;\left(t/\tau_{\mathrm{BODIPY}}^{\left(i\right)}\right),$$ (5)

where χ_*i*_ is the *i*th population fraction and $\tau_{\mathrm{BOD}}^{\left(i\right)}$ is the fluorescence lifetime of that population. Fluorescence decays from ZIP(C356)-lin-FKH and ZIP-lin-FKH(C493) in the absence and presence of DNA were analyzed using a double exponential model, obtaining the respective χ^2^_r_ to compare both models and to choose, using the F-test criteria, the statistically more robust behavior.

### Single-molecule anisotropy analysis

For each burst, we determine the steady-state anisotropy, *r*_*S*_, and the fluorescence weighted average lifetime, *τ*_*F*_, and compute two-dimensional frequency histograms referred to as anisotropy histogram. We describe populations on the anisotropy histograms by parametric relations.

For a single state with a rotational correlation time *ρ*, the integral steady-state anisotropy, *r*_*S*_, and the fundamental anisotropy *r*_0_ are related by the Perrin equation. For *N* states with different rotational correlation times, the equation becomes

$$r_{S}\left(\tau_{F}\right)=r_{0}\sum_{i=1}^{N}\frac{b_{i}}{1+\tau /\rho_{i}}.$$ (6)

We compute anisotropy lines for ${\left\langle \tau_{\mathrm{BOD}}\right\rangle }_{F}$ and *r*. In our single-molecule experiments, *r* was determined using the integrated background-corrected fluorescence intensities of the parallel *F*_*p*_ and perpendicular *F*_*s*_ detection channel,

$$r=\frac{F_{p}-F_{s}}{F_{p}+2GF_{S}},$$ (7)

where *G* is a factor correcting for differences in the detection efficiency of the *p* and *s* channels. We used the Perrin equation to compute the rotational correlation time, *ρ*_*i*_, and the population *x*_*i*_ of fluorophores in state *i*.

### smFCS

To determine the diffusion time *t*_*diff*_, all $G\left(t_{c}\right)$ curves were fitted with the model function [Eq. (8)] that considers a three-dimensional Gaussian confocal volume,

$$\begin{array}{ll} G\left(t_{c}\right) & =\frac{1}{N}\left(\frac{1}{1+\frac{t}{t_{\mathrm{diff}}}}\right)\sqrt{\left(\frac{1}{1+{\left(\frac{\omega }{z}\right)}^{2}\frac{t}{t_{\mathrm{diff}}\cdot z}}\right)}\\ & \;\times \left(1-\left|d\right|+\left|d\right|\;exp\;\left(-\frac{t}{t_{\mathrm{pho}}}\right)\right)+B, \end{array}$$ (8)

where N is the average number of particles in the confocal volume and ω and z are the axes for the geometrical volume. The *t*_*pho*_ term with amplitude $\left|d\right|$ is meant to account for the contribution of dye photophysical effects to the correlation and was allowed to vary for individual curves. *B* is a standard offset baseline for the calculated correlations.

## SUPPLEMENTARY MATERIAL

Two figures and six tables can be found in the supplementary material.

## Data Availability

The data that support the findings of this study are available from the corresponding authors upon reasonable request.
